# Highly Dispersed Ni Nanoclusters Spontaneously Formed on Hydrogen Boride Sheets

**DOI:** 10.3390/molecules27238261

**Published:** 2022-11-26

**Authors:** Natsumi Noguchi, Shin-ichi Ito, Miwa Hikichi, Yohei Cho, Kazuho Goto, Atsushi Kubo, Iwao Matsuda, Takeshi Fujita, Masahiro Miyauchi, Takahiro Kondo

**Affiliations:** 1 Graduate School of Pure and Applied Sciences, University of Tsukuba, Tsukuba 305-8574, Japan; 2 Faculty of Pure and Applied Sciences, University of Tsukuba, Tsukuba 305-8573, Japan; 3 School of Materials and Chemical Technology, Tokyo Institute of Technology, Meguro, Tokyo 152-8552, Japan; 4 Institute for Solid State Physics (ISSP), The University of Tokyo, Kashiwa 277-8581, Japan; 5 School of Environmental Science and Engineering, Kochi University of Technology, 185 Miyanokuchi, Tosayamada, Kami 782-8502, Japan; 6 R&D Center for Zero CO_2_ Emission Functional Materials and Tsukuba Research Center for Energy Materials Science (TREMS), University of Tsukuba, Tsukuba 305-8573, Japan; 7 The Advanced Institute for Materials Research, Tohoku University, Sendai 980-8577, Japan

**Keywords:** hydrogen boride (HB) sheets, Ni nanoclusters, transmission electron microscopy

## Abstract

Hydrogen boride (HB) sheets are two-dimensional materials comprising a negatively charged hexagonal boron network and positively charged hydrogen atoms with a stoichiometric ratio of 1:1. Herein, we report the spontaneous formation of highly dispersed Ni nanoclusters on HB sheets. The spontaneous reduction reaction of Ni ions by the HB sheets was monitored by in-situ measurements with an ultraviolet-visible spectrometer. Acetonitrile solutions of Ni complexes and acetonitrile dispersions of the HB sheets were mixed in several molar ratios (the HB:Ni molar ratio was varied from 100:0.5 to 100:20), and the changes in the absorbance were measured over time. In all cases, the results suggest that Ni metal clusters grow on the HB sheets, considering the increase in absorbance with time. The absorbance peak position shifts to the higher wavelength as the Ni ion concentration increases. Transmission electron microscopy images of the post-reaction products indicate the formation of Ni nanoclusters, with sizes of a few nanometers, on the HB sheets, regardless of the preparation conditions. These highly dispersed Ni nanoclusters supported on HB sheets will be used for catalytic and plasmonic applications and as hydrogen storage materials.

## 1. Introduction

Hydrogen boride (HB) sheets are two-dimensional (2D) nanosheets comprising a positively charged hydrogen and negatively charged boron network with an atomic ratio of B/H = 1.0. They were among the first synthesized hydrogenated borophenes (borophanes) [1]. Boron atoms form a hexagonal 2D network in HB sheets, wherein hydrogen atoms are bound to boron atoms by three-center two-electron (B–H–B) and two-center two-electron (B–H) bonds [2], with chemical stability against water [3]. HB sheets have been experimentally verified to exhibit excellent solid acid catalytic activity [4,5] and highly sensitive gas-sensing capability [2], as well as semimetal electronic [6], light-responsive hydrogen release [7], and carbon dioxide adsorption and conversion properties [8]. Furthermore, theoretical studies have revealed their intriguing electronic [9], optical, and thermal properties [10,11], as well as their possible applications in hydrogen release devices [12,13], reversible hydrogen storage [14], current limiters [15], photodetectors, individual amino acid sensors [16], rechargeable Li/Na ion battery electrodes [17,18], and anodes for rechargeable potassium-ion batteries, with high capacities, low voltages, and high rate performance [19]. Furthermore, the formation of HB sheets has paved the way for the conceptual development of new types of HB materials [20,21,22,23,24,25,26,27,28].

HB sheets also function as reductants for specific ions: the HB sheets had a redox potential between −0.277 and −0.257 V versus that of a standard hydrogen electrode (SHE) [29]. Therefore, metal nanoclusters can be supported by a single process, i.e., by mixing HB sheets with specific metal ions in a liquid [29], where metal ions with redox potentials lower than HB can be reduced, whereas those with redox potentials higher than HB cannot be reduced. This implies that only specific metals can be selectively reduced by HB sheets. By applying this property, HB sheets have been used to prepare superior composite electrode catalysts for oxygen evolution [30] and oxygen reduction reactions [31]. Therefore, a detailed understanding of the reduction properties of HB and the formation process of nanoclusters and/or nanocomposites is essential to promote the practical usage of HB sheets.

Herein, we report the spontaneous formation of highly dispersed Ni nanoclusters on HB sheets. Ni nanoclusters were formed on HB sheets by mixing Ni ions and HB sheets in an acetonitrile solution at different Ni ion concentrations. Although the sizes of the Ni nanoclusters were not significantly affected by the concentration of Ni ions, their densities were controlled by the concentration. The resulting highly dispersed Ni nanoclusters supported on the HB sheets may be useful for catalytic and plasmonic applications, as well as hydrogen storage materials [32,33].

## 2. Results and Discussion

**Figure 1:**
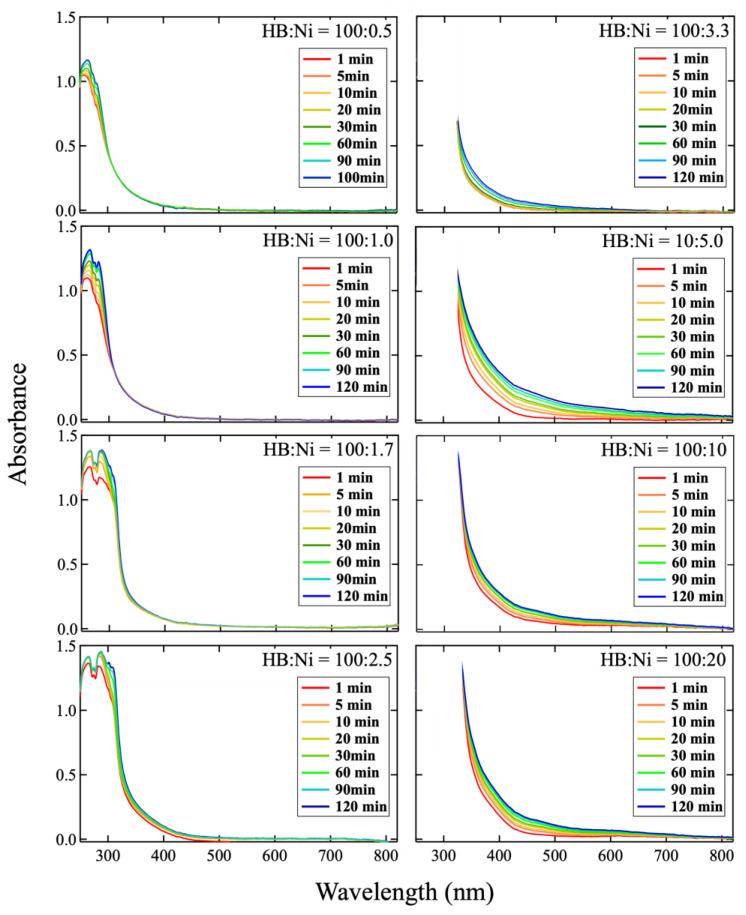
Temporal change in UV-vis spectra of HB dispersion at 0.02 mol/L in acetonitrile after mixing with Ni ions at various Ni ion concentrations. Molar ratio of the HB:Ni ions was adjusted from 100:0.5 to 100:20. Spectrum range under 330 nm for HB:Ni = 100:3.3, 100:5.0, 100:10, and 100:20 represents the range of intensity saturation, and is not shown here. Legend indicates time (1, 5, 10, 20, 30, 60, 90, and 120 min) elapsed from the start of mixing the two dispersions.

**Figure 2:**
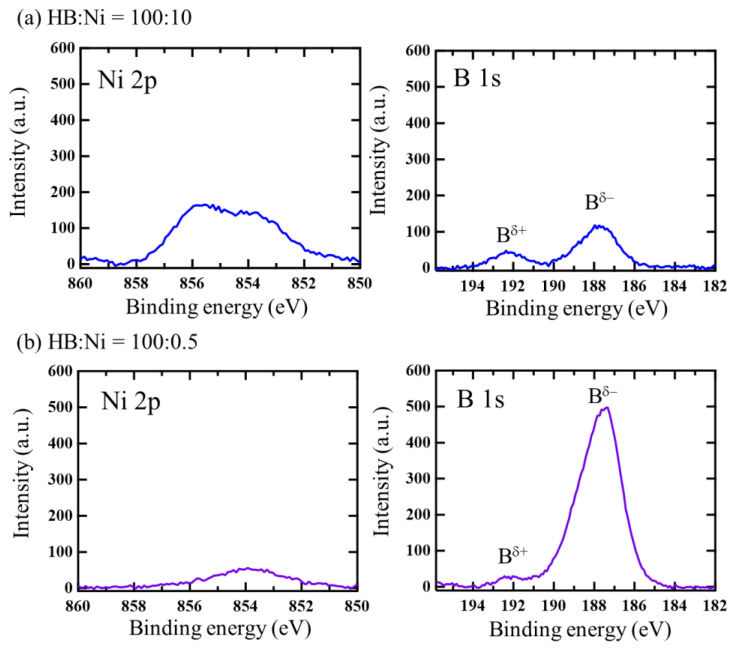
XPS results of the sample after drying the following mixtures in acetonitrile: (**a**) HB:Ni = 100:10, and (**b**) HB:Ni = 100:0.5.

Figure 1 shows the temporal change in the ultraviolet-visible (UV-vis) spectra of the HB dispersion at a 0.02 mol/L concentration in acetonitrile after mixing with Ni ions at various Ni ion concentrations (the molar ratio of the HB:Ni ions was adjusted from 100:0.5 to 100:20). The spectra shorter than 250 nm are not shown, since their photon numbers in a UV-Vis detector were too low for quantitative comparison (Figure S1). Moreover, the spectrum shorter than 330 nm for the high concentrated Ni ions conditions are not shown because the original absorbance intensities are too high to be saturated in this range. Across all concentrations, after mixing, the absorbance increases with time, at specific wavenumbers. This change can be attributed to the increase in the plasmon absorbance of the Ni nanoclusters on the HB sheets because of the spontaneous reduction reaction of the Ni ions on the HB sheets. In this reaction, the Ni ions may be exchanged with the protons of the HB sheets, and the electrons in the boron species would reduce the Ni ions, resulting in the formation of metallic Ni nanoclusters onto the HB sheets, while positively charged B would locally appear, similar to the results for Cu nanoclusters formed on HB sheets [29]. To evaluate the chemical states of Ni and B in the products, X-ray photoelectron spectroscopy (XPS) was performed for the samples of HB:Ni = 100:0.5 and 100:10, as shown in Figure 2. In both cases, the Ni peak and B^δ+^ component are detected, along with the B^δ−^ component, which originated from the pure HB sheets (for comparison, the XPS result for the pristine HB sheets is shown in Figure S2). The Ni intensity and B^δ+^ population are larger for HB:Ni = 100:10 than those for HB:Ni = 100:0.5, and a consistent correlation between the B:Ni ratio and B^δ+^/B^δ−^ is obtained (Table 1), indicating that Ni deposition occurs because of the spontaneous reduction reaction of the following process:

nH^+^B^−^ + Ni^2+^ → 2H^+^ + Ni^2+^·(n − 2)H^+^nB^−^
 → 2H^+^ + Ni·(n − 2)H^+^(n − 1)B^−^·B^+^ (1)

It should be noted that the appearance of oxide Ni peak for the sample with HB:Ni = 100:10, shown in Figure 2a, may arise from the surface oxidation of Ni nanoclusters because of the need to expose the sample to air before setting it in the XPS load-lock chamber. A similar spontaneous reduction reaction has also been reported in the formation of nanocomposites using graphene [34], layered CaSi_2_ [35], polysilane [36], and Mg-deficient hydroxyl-functionalized boron nanosheets [37].

**Figure 3:**
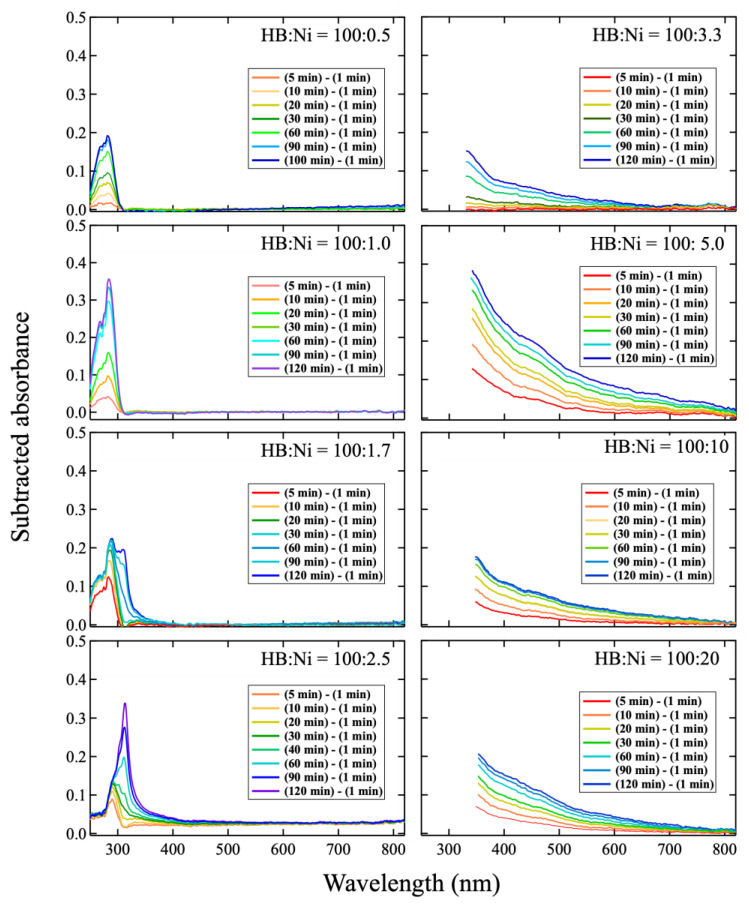
Subtracted spectra from UV-vis measurement; each spectrum shows a difference immediately after mixing (1 min). For the sample of HB:Ni = 100:2.5, the subtracted spectrum of “(40 min)-(1 min)” is also presented because it shows multiple components.

**Figure 4:**
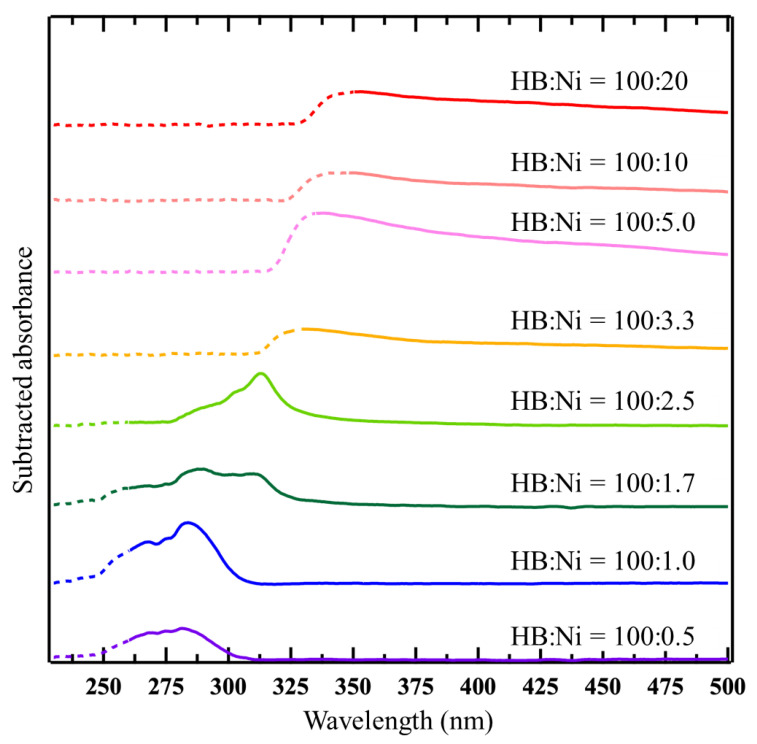
Respective subtracted spectra of HB:Ni = 100:20 (90 min), 100:10 (120 min), 100:5.0 (120 min), 100:3.3 (120 min), 100:2.5 (120 min), 100:1.7 (120 min), 100:1.0 (120 min), and 100:0.5 (120 min). The wavelength ranges for the intensity saturation in the original spectra and/or low detection sensitivity are shown by dots.

In Figure 1, the wavelength of the absorbance changes according to the concentration of Ni ions. To study the change, each spectrum was subtracted by the spectrum captured at 1 min after mixing, as shown in Figure 3. In the case of a lower Ni concentration (HB:Ni = 100:0.5, HB:Ni = 100:1.0, and HB:Ni = 100:1.7), the absorbance in the range of 250–300 nm increases with time after mixing. For a higher Ni ion concentration (HB:Ni = 100:2.5, HB:Ni = 100:3.3, HB:Ni = 100:5.0, HB:Ni = 100:10, and HB:Ni = 100:20), the absorbance ranging from 320 to 800 nm increases with time after mixing. The change in the absorption feature as a function of the Ni ion density is highlighted in Figure 4, where a representative subtracted spectrum when the spectrum change is saturated is shown. The absorbance peak position shifts to the longer wavelength as the Ni ion concentration increases. 

Two possible reasons account for the shift in the absorbance wavelength: (1) the sizes of the Ni nanoclusters formed on the HB sheets increase in proportion to the Ni ion concentration, and (2) the effect of localized surface plasmon resonance due to the change in the distance between the Ni nanoclusters on the HB sheets. Regarding (1), it has been reported that the absorption peak due to surface plasmon resonance shifts to longer wavelengths as the particle size of the metal nanoclusters increases [38,39,40]. For (2), the absorption peak shifts to longer wavelengths because of surface plasmon coupling when the distance between the nanoclusters decreases [41]. 

**Figure 5:**
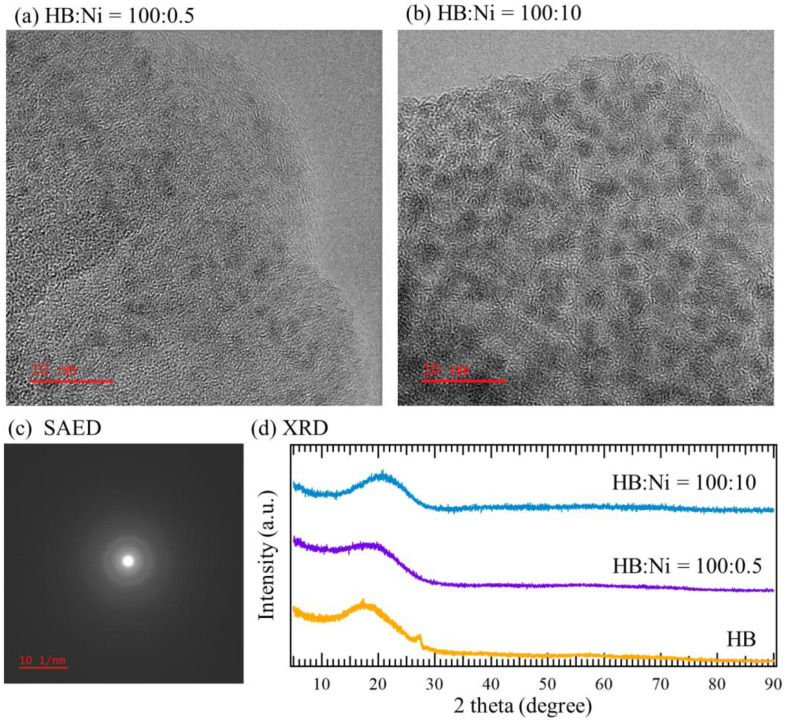
TEM images SAED pattern and XRD patterns. (**a**) TEM images of HB:Ni = 100:0.5, and (**b**) HB:Ni = 100:10. (**c**) SAED pattern simultaneously obtained with the TEM image shown in panel b. (**d**) XRD patterns of HB, HB:Ni = 100:0.5, and HB:Ni = 100:10 (a Kapton capsule was used as a cover of the sample to prevent the exposure of the sample to the atmosphere). The XRD pattern of HB shows a small peak at 27°, which is considered to be originated from the stacking of some of the sheets.

To clarify which of the two factors caused the shift in the absorption peaks in this study, samples were prepared with two distinctly different Ni ion concentrations (HB:Ni = 100:10 and 100:0.5, 42 d and 48 d after mixing) and observed via transmission electron microscopy (TEM); the results are shown in Figure 5. In both samples, several clusters are observed as dark spheres, suggesting that Ni atoms form clusters after the reduction reaction of Ni ions on the HB sheets, as reported previously [29]. Indeed, the presence of Ni with B is clearly seen in the obtained energy dispersive X-ray spectroscopy (EDS) conducted during TEM (Figure S3), which is consistent with the XPS results (Figure 2). The selected area electron diffraction (SAED), which was conducted during TEM, shows a halo pattern, as seen in Figure 5c. This indicates that the size of the Ni nanoclusters is considerably small. Consistently, X-ray diffraction (XRD) patterns show no diffraction peaks, indicating that there are no larger sized Ni particles in the sample. 

**Figure 6:**
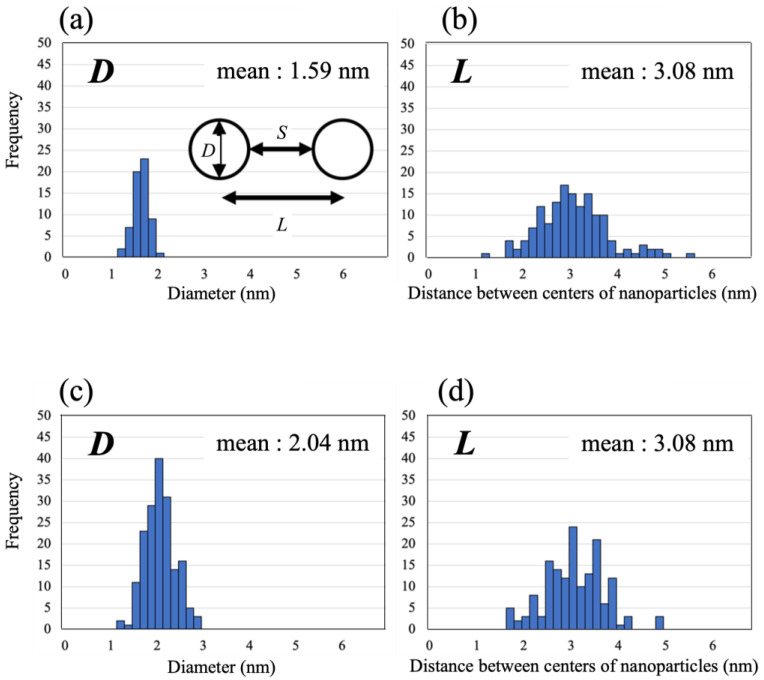
Histograms of (**a**,**c**) diameter, (**b**,**d**) distance between centers of nanoparticles. (**a**,**b**) are the results at HB:Ni = 100:0.5, (**c**,**d**) are the results at HB:Ni = 100:10.

To quantitatively evaluate the size and dispersion of the Ni nanoclusters on the HB sheets, the diameter (*D*) and inter-center distance (*L*) of the dark spheres in 17 TEM images were statistically analyzed, as shown by the histogram in Figure 6. The mean *D* values of the Ni nanoclusters for HB:Ni = 100:0.5 and 100:10 were estimated as 1.59 ± 0.02 and 2.04 ± 0.03 nm, respectively, and the mean values of *L* were approximately 3.08 ± 0.06 and 3.08 ± 0.05 nm, respectively. This result indicates that the sizes of the Ni nanoclusters formed on the HB sheets do not vary significantly with the Ni ion concentration. This contrasts with the case of Pt clusters formed on graphene using a similar method, where the sizes of the Pt clusters were reported to vary proportionally to the amount of prepared Pt precursors [42]. In other words, HB can easily support highly-dispersed small metal nanoclusters with high density, in contrast to graphene. In this study, the Ni ions presumably interact strongly with the HB sheets when they are reduced. The Ni clusters are then considered to be anchored at the reacted sites on the HB sheets, preventing significant aggregation, which differs from the metal ions reduction process using a reducing agent under homogeneous media.

From the average values of *L* and *D*, the inter-surface distance (*S*, the length between the nearest cluster surfaces) values can be derived as 1.04 ± 0.06 nm for HB:Ni = 100:10, and 1.49 ± 0.07 nm for HB:Ni = 100:0.5. Notably, as the particle size increases, due to plasmon resonance, the peak position of the spectrum shifts toward longer wavelengths [38,39,40,43]. For example, in the case of Au clusters, a change in the particle size from 9 to 99 nm results in a peak shift of 25 nm [43]. Conversely, the spectral peak position shifts by approximately 40 nm when the interparticle distance of the Au clusters changes from 12 to 6 nm [41]. Previous studies suggest that the plasmon resonance is more sensitive to the interparticle distance than to the particle size. As shown in Figure 4, the optical absorption peak shifts 56 nm from HB:Ni = 100:0.5 to HB:Ni = 100:10, where the difference in *D* of these samples is 0.45 ± 0.03 nm and that of *S* is 0.45 ± 0.09 nm. Based on the previous plasmonic studies, the present optical absorption shift is ascribed to the change in the interparticle distance *S*, rather than particle size *D*. This peak shift is also observed during the nanoparticle formation for HB:Ni = 100:2.5, as shown in Figure 3 (the main peak position shifts from 290 to 320 nm).

The resultant highly dispersed Ni nanoclusters supported on the HB sheets may be useful for catalytic and plasmonic applications, and also as hydrogen storage materials, because highly dispersed Ni-clusters have been reported to contribute to the superior hydrogen desorption kinetics of MgH_2_ [32,33].

## 3. Materials and Methods

### 3.1. Materials

MgB_2_ powder (99%, RareMetallic Co., Ltd., Tokyo, Japan), acetonitrile (99.5%,Wako Pure ChemicalIndustries Ltd., Osaka, Japan), and an ion-exchange resin (AmberliteIR120B hydrogen, Organo Corp., Tokyo, Japan) were used to synthesize the HB sheets and the HB dispersion. Ni(C_5_H_7_O_2_)_2_ (98%, Merck) was used to prepare the Ni(C_5_H_7_O_2_)_2_ dispersion.

### 3.2. Synthesis of HB Sheets

The HB sheets were prepared using a previously reported ion-exchange method [1]. MgB_2_ (1.0 g) powder in acetonitrile (200 mL) was added to the ion-exchange resin (60 mL) in acetonitrile (200 mL) in a Schlenk flask under a nitrogen atmosphere. The mixture was stirred with a magnetic stirrer at 310 rpm for 3 d at room temperature (~300 K). The process was sensitive to water because of the hydrolysis reaction of MgB_2_ [44]. Thus, water was carefully removed beforehand. In this study, the recently reported acid-assisted reaction was not applied [45]. The supernatant was kept for 1 d at 255 K to solidify and separate the byproduct B(OH)_3_. Dried HB sheets were prepared by heating the resulting liquid at 343 K while pumping with a cooling trap. We characterized the product rigorously using X-ray photoelectron spectroscopy (XPS) to confirm the absence of Mg, the presence of negatively charged B, and the absence of oxidized B, as shown in Figure S2 [1,2,3,4,5,6,7,8,29].

### 3.3. XPS Measurements

To characterize the sample, XPS measurement was performed using a JPS 9010 TR spectrometer (XPS; JPS 9010 TR, JEOL, Ltd., Tokyo, Japan) equipped with an ultrahigh vacuum chamber and an Mg Kα X-ray source (1253.6 eV). The pass energy was 10 eV, the energy resolution (estimated from the Ag 3d5/2 peak width of a clean Ag sample) was 0.635 eV, and the binding energy uncertainty was ±0.05 eV. The sample was placed on a graphite tape. The Shirley background was subtracted from the spectrum using SpecSurf version 1.8.3.7 (JEOL, Ltd., Japan). The same software was used for estimating the quantitative atomic ratio of Ni and B of the reaction product, based on the peak area and sensitivity factors. The charge build-up in the sample (because of the incomplete contact of the graphite tape with the sample holder) resulted in a slight shift to higher binding energies for those spectra. Therefore, we calibrated the charge build-up based on the C1s peak of the graphite tape as 284.6 eV. The charge states of boron were judged based on the B 1s core level peak position by comparing the results with those in the literature (listed in the Supplementary Information of Ref. [46]).

### 3.4. UV-vis Measurements

A UV-vis spectroscope (DH-2000-BAL, Ocean Optics, Inc., Dunedin, FL, USA) was used to measure the absorbance of the Ni(C_5_H_7_O_2_)_2_ and HB dispersions. For this, Ni(C_5_H_7_O_2_)_2_ powder was dispersed in acetonitrile at concentrations of 0.04, 0.002, 0.001, 7 × 10^−4^, 5 × 10^−4^, 3 × 10^−4^, 2 × 10^−4^, and 1 × 10^−4^ mol/L. The HB sheets (powder, 0.0059 g) were dispersed into acetonitrile (25 mL) at a fixed concentration of 0.02 mol/L. The absorbance of the mixture of the Ni(C_5_H_7_O_2_)_2_ dispersion and HB dispersion was then measured using the UV-vis spectrometer. The intensity of the light source of the UV-vis device was measured beforehand (Figure S1) to obtain a reliable wavelength range for the UV-vis measurements. To study the change in the UV-vis spectra, the subtracted spectra were used, as reported previously [29], where each spectrum was subtracted by the spectrum that was captured at 1 min after mixing.

### 3.5. TEM, SAED, and EDS Measurements

Measurements were performed at room temperature using a JEM-2100F TEM/STEM apparatus (JEOL, Ltd., Japan) with double spherical aberration (Cs) correctors (CEOS GmbH, Heidelberg, Germany) to obtain high-contrast images with a point-to-point resolution of 1.4 Å. The lens aberrations were optimized by evaluating the Zemlin tableau of amorphous carbon. The residual spherical aberration was almost zero (Cs = −0.8 ± 1.2 μm with 95% certainty). The acceleration voltage was set to 120 kV, which is the lowest voltage that is effective with the Cs correctors in this system.

### 3.6. XRD Measurements

The XRD patterns were recorded at room temperature (~300 K) using a benchtop X-ray diffractometer (Rigaku MiniFlex, Tokyo, Japan), which employed Cu Kα radiation. The X-rays were generated using the line focus principle. A reflection-free Si plate was used as the sample stage. The Kapton capsule was used as a cover for the sample to prevent the exposure of the sample to the atmosphere. The diffraction patterns were recorded using a D/teX Ultra silicon strip detector (Rigaku) at 0.01° s^−1^ up to a 2θ value of 90°.

## 4. Conclusions

When a solution of Ni ions was mixed with an HB dispersion, it was confirmed that the Ni ions were reduced to form Ni nanoclusters on the HB sheets, without the use of reduction reagents. The TEM images showed that the Ni nanoclusters were small (1–2 nm) and highly dispersed, without aggregation. This was ascribed to the anchoring effect due to the strong interaction between the reduced Ni and HB sheets. By changing the concentration of Ni ions, the density of the particles could be changed without significantly changing the size of the particles. These highly dispersed Ni nanoclusters on HB sheets are expected to be used as catalysts and hydrogen storage materials, as well as in plasmonic applications.

## Figures and Tables

**Table 1:** Analyzed results of XPS spectra.

	Atomic Ratio Estimated	B 1s Peak	
Sample	from Peak Area (B:Ni)	B^δ−^	B^δ+^
HB:Ni = 100:0.5	100:0.5	96%	4%
HB:Ni = 100:10	100:5.4	67%	33%

## Data Availability

Data is available on request from the corresponding authors.

## Supplementary Materials

The following supporting information can be downloaded at: https://www.mdpi.com/article/10.3390/molecules27238261/s1, Figure S1: Intensity of the light source of the UV-vis device; Figure S2: XPS of HB; Figure S3: Energy-dispersive X-ray spectroscopy (EDS).
