# Supplementary material for: Highly Dispersed Ni Nanoclusters Spontaneously Formed on Hydrogen Boride Sheets
Source: Molecules. 2022 Nov 26;27(23):8261. doi: 10.3390/molecules27238261 (PMC9736194; doi:10.3390/molecules27238261)
Supplement: Supplementary file 1 [file molecules-27-08261-s001.zip › molecules-2011691-supplementary.pdf]

Supplementary Information

Highly dispersed Ni nanoclusters spontaneously formed on hydrogen boride sheets

Natsumi Noguchi[1], Shin-ichi Ito[2], Miwa Hikichi[2], Yohei Cho[3], Kazuho Goto[1], Atsushi Kubo[2], Iwao Matsuda[4], Takeshi Fujita[5], Masahiro Miyauchi*[3], and Takahiro Kondo*[2,6,7]

[1]Graduate School of Pure and Applied Sciences, University of Tsukuba, Tsukuba 305-8574, Japan

[2]Faculty of Pure and Applied Sciences, University of Tsukuba, Tsukuba 305-8573, Japan

[3]School of Materials and Chemical Technology, Tokyo Institute of Technology, Meguro 152-8552, Japan

[4]Institute for Solid State Physics (ISSP), The University of Tokyo, Kashiwa, 277-8581, Japan

[5]School of Environmental Science and Engineering, Kochi University of Technology, 185 Miyanokuchi, Tosayamada, Kami 782-8502, Japan

[6]R&D Center for Zero CO_2 Emission Functional Materials and Tsukuba Research Center for Energy Materials Science (TREMS), University of Tsukuba, Tsukuba 305-8573, Japan

[7]The Advanced Institute for Materials Research, Tohoku University, Sendai 980-8577, Japan.

*mmiyauchi@ceram.titech.ac.jp, (M. Miyauchi), *takahiro@ims.tsukuba.ac.jp (T. Kondo)

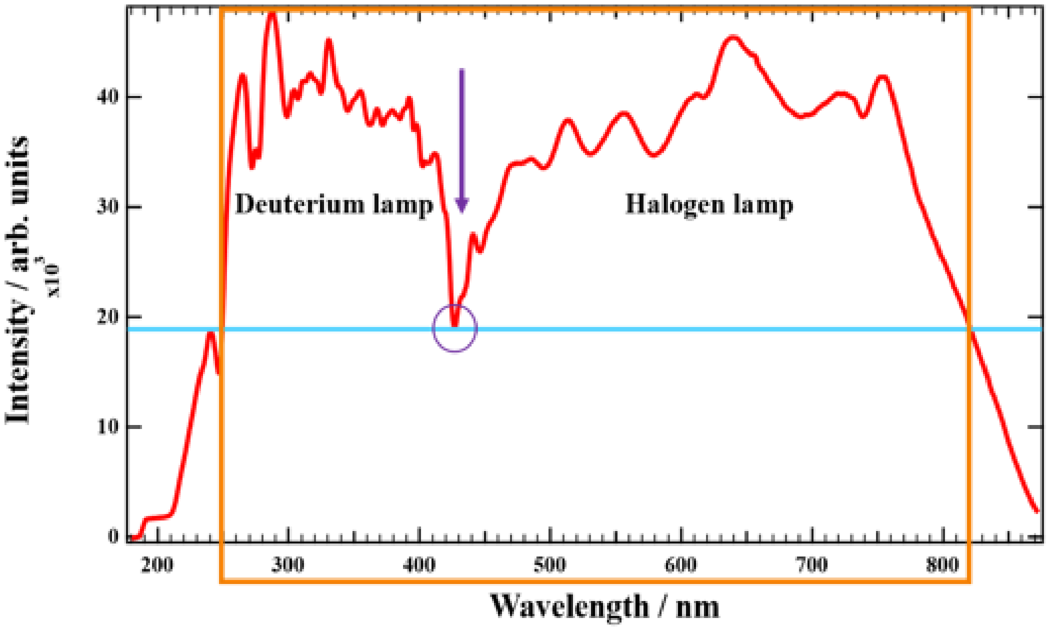

Figure S1 Intensity of the light source of the UV-vis device. Areas with intensity less than that of the border between the deuterium lamp and the halogen lamp (below 250 nm and above 820 nm) were considered unreliable because of the low intensity of the light source.

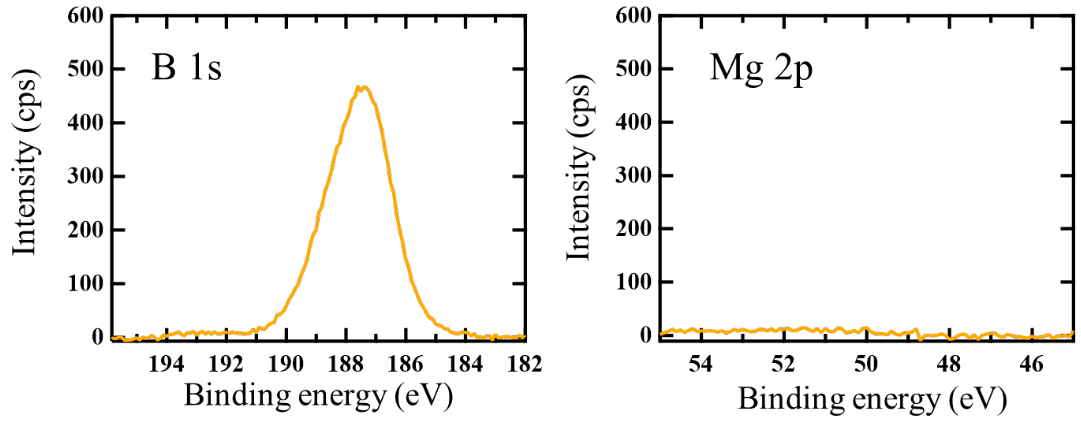

Figure S2 X-ray photoelectron spectra of HB. Absence of Mg, presence of negatively charged B, and absence of oxidized B indicate that HB does not include the by-product $B(OH)_3$, as well as starting material MgB_2 [1-8,29].

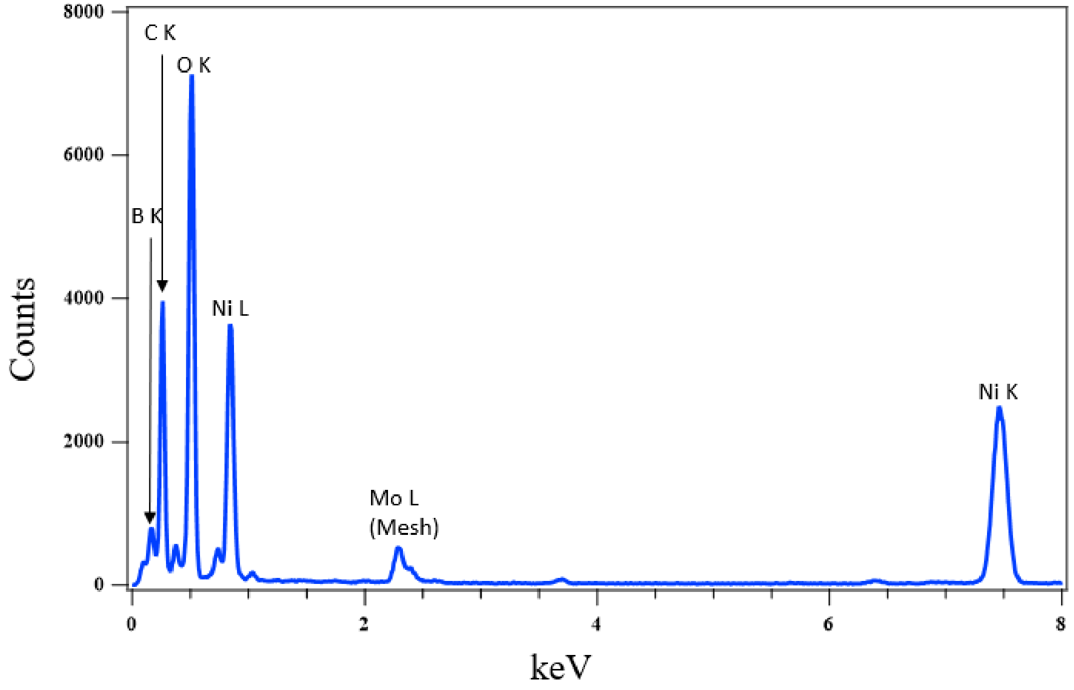

Figure S3 Energy-dispersive X-ray spectra (EDS). EDS of Ni nanoclusters supported HB (HB:Ni = 100:10). B of HB, C, O, and Ni are observed (intensity of B is small because the sensitivity for B is low).
